# DNA Protection against Oxidative Damage Using the Hydroalcoholic Extract of* Garcinia mangostana* and Alpha-Mangostin

**DOI:** 10.1155/2016/3430405

**Published:** 2016-03-06

**Authors:** Ronaldo Carvalho-Silva, Alanna Cibelle Fernandes Pereira, Rúbens Prince dos Santos Alves, Temenouga N. Guecheva, João A. P. Henriques, Martin Brendel, Cristina Pungartnik, Fabrício Rios-Santos

**Affiliations:** ^1^Laboratório de Biologia de Fungos, Centro de Biotecnologia, Universidade Estadual de Santa Cruz (UESC), Rodovia Jorge Amado, km 16, 45662900 Ilhéus, BA, Brazil; ^2^Laboratório de Farmacogenômica e Epidemiologia Molecular, Universidade Estadual de Santa Cruz (UESC), Rodovia Jorge Amado, km 16, 45662900 Ilhéus, BA, Brazil; ^3^Laboratório de Desenvolvimento de Vacinas, Instituto de Ciências Biomédicas, Departamento de Microbiologia, Universidade de São Paulo, São Paulo, SP, Brazil; ^4^Genotox-Royal, Universidade Federal do Rio Grande do Sul (UFRGS), Avenida Bento Gonçalves, 9500/Setor 4-Pred 43.421/S113, 91501970 Porto Alegre, RS, Brazil; ^5^Laboratório de Imunofarmacologia, Universidade Federal de Mato Grosso (UFMT), Avenida Fernando Corrêa da Costa, n° 2367, Boa Esperança, 78060900 Cuiabá, MT, Brazil

## Abstract

*Garcinia mangostana,* popularly known as “mangosteen fruit,” originates from Southeast Asia and came to Brazil about 80 years ago where it mainly grows in the states of Pará and Bahia. Although mangosteen or its extracts have been used for ages in Asian folk medicine, data on its potential genotoxicity is missing. We, therefore, evaluated genotoxicity/mutagenicity of hydroethanolic mangosteen extract [HEGM, 10 to 640 *μ*g/mL] in established test assays (Comet assay, micronucleus test, and* Salmonella*/microsome test). In the Comet assay, HEGM-exposed human leukocytes showed no DNA damage. No significant HEGM-induced mutation in TA98 and TA100 strains of* Salmonella typhimurium* (with or without metabolic activation) was observed and HEGM-exposed human lymphocytes had no increase of micronuclei. However, HEGM suggested exposure concentration-dependent antigenotoxic potential in leukocytes and antioxidant potential in the yeast* Saccharomyces cerevisiae*. HEGM preloading effectively protected against H_2_O_2_-induced DNA damage in leukocytes (Comet assay). Preloading of yeast with HEGM for up to 4 h significantly protected the cells from lethality of chronic H_2_O_2_-exposure, as expressed in better survival. Absence of genotoxicity and demonstration of an antigenotoxic and antioxidant potential suggest that HEGM or some substances contained in it may hold promise for pharmaceutical or nutraceutical application.

## 1. Introduction

Despite many biological activities attributed to medicinal plants and their pharmacological properties, a minor contribution exists in regard to their safety and toxicity in humans [1]. The indiscriminate use of medicinal plants or natural products in traditional medicine or its self-administration in combination with prescribed drugs could, therefore, cause serious therapeutic problems, that is, leading to unknown deleterious effects by the plant product or severe adverse drug cross effects, respectively [2, 3]. It is worthwhile to mention that, apart from acute toxicity of plant effects, for example, carcinogenic potential, are more difficult to evaluate, as it will be manifested only after significant time after initial exposure. Thus, it is not only important to describe the pharmacological properties of a medicinal plant but also to establish its safety for human consumption.

The fruit of* Garcinia mangostana* L., a tree originating from Southern Asia, is widely consumed in Brazil, particularly in its northwest region, as it has not only an excellent taste but also is supposed to have a range of medicinal properties, that is, in treating constipation, diarrhea, intestinal disorders, skin diseases, and cancer [4, 5]. In fact,* G. mangostana* extract was shown to have antitumor, anti-inflammatory, antiallergy, antibacterial, antifungal, antiviral, and antimalarial activity [5]. Most biological properties of* G. mangostana* extracts may be related to xanthones, mainly isolated from the pericarp, whole fruit, and bark or from leaves. Phytochemical analysis of* G. mangostana* revealed a significant variety of xanthones, mainly alpha-, beta-, and gamma-mangostins, garcinone E, 8-deoxygartanin, and gartanin [5]. While chemical studies of these compounds have progressed, only limited data is available on their cytotoxicity, either when applied as a mixture contained in* G. mangostana* extracts or as one of its purified chemical compounds.

*In vivo* toxicity studies established a lethal dose at 1,000 mg/kg for female BALB/c mice and suggested a suitable dose for short-term studies of less than 200 mg/kg [6]. A few cytotoxicity studies are available: mouse xenograph anticancer testing showed xanthones to inhibit neoplastic cell reproduction [7]; anticolon cancer effect was investigated on HCT116 human colorectal carcinoma cells including cytotoxicity, apoptosis, antitumorigenicity, and effect on cell signaling pathways; dose dependent killing of HCT116 cells exposed to mangosteen xanthone extract, *α*-mangostin, and *γ*-mangostin gave IC_50_ values of 6.5 ± 1.0 *μ*g/mL, 5.1 ± 0.2 *μ*g/mL, and 7.2 ± 0.4 *μ*g/mL, respectively; finally, *α*-mangostin treatment induced mitochondria-mediated toxicity and apoptosis in human breast cancer line MDA-MB231 [8].

*G. mangostana* may contain various compounds which, when extracted, could have beneficial biological activities and thus could be a source for pharmaceutical and nutraceutical products similar to those already found in* Teucrium ramosissimum* [9],* Copaifera langsdorffii* [10], and* Moringa oleifera* [11]. Unfortunately, data on genotoxicity of crude extract as well as substances extracted from mangosteen pericarp is not available. This leaves a gap in our knowledge on the anticancer potential of mangosteen extract and on mangostin safety practiced in self-medication in traditional medicine [5].

In this work we, therefore, evaluated the genotoxicity/mutagenicity and the antigenotoxic potential of the hydroethanolic extract of* G. mangostana* [HEGM] in a combination of biological tests, that is, micronucleus, Comet assay, Ames test, and antioxidant activity in the yeast* Saccharomyces cerevisiae*.

## 2. Materials and Methods

### 2.1. Preparation of Extract

Fruits of* Garcinia mangostana* L. were collected at Una, Bahia, Brazil. After cleaning and washing, the pericarp of fruits (approximately 20 g) was broken and macerated for 24 h with 1 L of ethanol (70%). The supernatant was vacuum-filtered (filter paper) and concentrated by rotary evaporation until complete withdrawal of ethanol, followed by freezing and lyophilization to obtain the hydroethanolic extract of* G. mangostana* [HEGM]. In all experiments HEGM was dissolved with DMSO (7 mM).

All media and chemicals used are SIGMA (Sigma-Aldrich, Inc., St. Louis, MO, USA) or VETEC (Vetec Quimica Fina Ltda, RJ, Brazil) or as otherwise stated.

### 2.2. HEGM Cytotoxicity

Human blood cell viability test by trypan blue exclusion was used to determine adequate concentrations of HEGM. Blood was collected from a healthy individual. Samples of 20 *μ*L of whole blood/mL of RPMI 1640 medium contained HEGM at doses ranging from 10 to 1280 *μ*g/mL. Cell viability was measured by collecting HEGM-exposed samples after 1, 2, and 4 h of incubation at 37°C and mixing 15 *μ*L of each cell suspension with 15 *μ*L of trypan blue (4%) in a microcentrifuge tube. Cell suspension was analyzed by light microscopy in a Neubauer chamber. Viable cells were unstained while dead cells appeared stained blue. HEGM doses used in this study allowed cell viability >90%. Total 2,100 cells were analyzed throughout the experiment. Through the line equation of the IC_50_ was IC_50_ 1853 *μ*g/mL.

### 2.3. HEGM Genotoxic Activity

#### 2.3.1. Comet Assay

Nucleated cells from peripheral blood were obtained from 8 mL of human peripheral blood of a male, age 26, and nonsmoking healthy individual. Blood was transferred to tubes containing anticoagulant and centrifuged for 10 min at 1000 g. The buffy coat was collected along with red blood cells and serum. Cells were then transferred to a sterile microcentrifuge tube and stored protected from light at room temperature until the start of microcultivations.

The Comet assay was performed based on the protocol proposed by Tice et al. [12]. Briefly, a cell suspension of 20 *μ*L of cell culture in 1 mL of RPMI 1640 was exposed to 20 *μ*L of HEGM (final concentration of 10 to 640 *μ*g/mL, 1 h, 37°C), DMSO as negative control (7 mM, 1 h, 37°C), or hydrogen peroxide as positive control (H_2_O_2_, 1 mM, 1 h, 37°C); 15 *μ*L of that cell suspension was mixed with 95 *μ*L of low-melting agarose in a microcentrifuge tube. The cell suspension was transferred to an agarose precoated glass slide, covered with a glass cover slip (22 mm × 66 mm), and immediately placed in a refrigerator (4°C) for 5 min to allow complete agarose solidification. Slides were prepared in duplicate. Cover slips were then removed and the slides immersed in cold (4°C) lysing solution (2.5 M NaCl, 100 mM Na_2_EDTA, 10 mM Tris, 1% (v/v) Triton X-100, and 10% DMSO, pH 10), protected from light for at least 8 h. DNA was allowed to unwind for 20 min in freshly prepared alkaline electrophoresis buffer (1 mM Na_2_EDTA, 0.3 N NaOH, pH 13) prior to electrophoresis. Horizontal electrophoresis was performed at 25 V/cm for 20 min at 4°C. All steps were performed under yellow light to minimize additional DNA damage. The slides were then placed vertically and gently washed 3x for 5 min with neutralizing buffer (0.4 M Tris-HCl buffer, pH 7.5).

Slides were dried at 37°C for 1.5 h and then fixed for 10 min (15% trichloroacetic acid, 5% zinc sulphate heptahydrate, and 5% glycerol), washed 3 times with distilled water. After 24 h fixation, the slides were stained with silver nitrate (1%) in a rotatory shaker for 20 min at 37°C. Slides were washed 3 times with distilled water, neutralized with acetic acid (1%) for 5 min, and again washed 3x with distilled water. The slides were then dried at room temperature and properly stored until analysis.

Two persons analyzed each sample twice by optical microscopy (100-fold magnification). In each of the two slides, 100 Comets were analyzed and classified as 0, 1, 2, 3, and 4, according to the size of their tail type [12]. The index of genetic damage at each HEGM concentration was determined by summing the number of multiplications of Comets with their respective class and the percentage of damage in each treatment.

Bars in the Comet assay represent mean and SD of three independent experiments (values obtained from an average of 100 cells per pool). ANOVA-Tukey was conducted to compare means; bars represent mean ± SD as calculated by the GraphPad Prism® program (GraphPad Software, Inc., San Diego, CA).

#### 2.3.2. Salmonella/Microsome Mutagenicity Assay (Ames Test)

*Salmonella typhimurium* strains TA98 and TA100 were kindly provided by B. M. Ames (University of California, Berkeley, CA, USA). Mutagenicity was assayed by the preincubation procedure. The S9 metabolic activation mixture (S9 mix) was prepared according to Maron and Ames [13]. Briefly, 100 *μ*L of bacterial test cultures (1-2 × 10^9^ cells/mL) was incubated for 20 min, at 37°C with different amounts of* G. mangostana* extract in the presence or absence of S9 mix without shaking. Subsequently, 2 mL of soft agar (0.6% agar, 0.5% NaCl, 50 *μ*M histidine, and 50 *μ*M biotin, pH 7.4, 46°C) were added to the test tube and poured immediately onto a plate of minimal agar (1.5% agar, Vogel-Bonner E medium, containing 2% glucose). The concentration range of HEGM extract was determined in experiments using* S. typhimurium* strain TA100, with and without metabolization, and cytotoxicity was observed at concentrations higher than 20 *μ*g/plate in the absence of S9 mix and 500 *μ*g/plate in the presence of S9 mix. In the mutagenicity assay, the dose range between 100 and 500 *μ*g/plate was used in the presence of S9 mix and a concentration range between 4 and 20 *μ*g/plate was applied in the absence of S9 mix. Aflatoxin B1 (1 *μ*g/plate) was used as positive control for all strains in the presence of metabolic activation (+S9 mix). In the absence of metabolic activation, 4-nitroquinoline-oxide (4-NQO, 0.5 *μ*g/plate) was used for strain TA98 and sodium azide (1 *μ*g/plate) for strain TA100. Plates were incubated in the dark at 37°C for 48 h before counting the revertant colonies.

The results were analyzed by the* Salmonella* Statistic Assay (Environmental Monitoring System Laboratory, EPA, Software Version 2.3, April 1988). A test substance was considered mutagenic when significant dose response and ANOVA variance were observed, and the increase in the mean number of revertants on test plates was at least twofold higher than that observed in the negative control.

#### 2.3.3. Micronucleus Test (MN)

The modified cytokinesis blocked method of Fenech and Morley [14] was used to determine the frequency of MN. Briefly, 1 mL of whole blood was mixed with 9 mL of RPM1 1640, 10% fetal bovine serum, penicillin (100 units/mL), and streptomycin (100 *μ*g/mL). Phytohemagglutinin was added at a concentration of 2% to stimulate lymphocyte division. Cells were exposed to concentrations of HEGM (160 to 640 *μ*g/mL) and incubated in 5% CO_2_, at 37°C for 72 h. Vincristine (1 nM) and incubation in 5% CO_2_ at 37°C for 72 h were used as positive control whereas DMSO (7 mM) and incubation in 5% CO_2_ at 37°C for 72 h were the negative control. At 44 h, cytochalasin B was added at a concentration of 3 *μ*g/mL. At 72 h cells were collected by centrifugation at 1,000 rpm for 10 min and then treated with 1% of sodium citrate hypotonic solution for 5 min at room temperature. Treatment with methanol/acetic acid (3 : 1, v/v) was repeated twice, and the cells were collected on microscope slides. Slides were coded, stained with 5% Giemsa for 5 min and scored according to the criteria described [14]. The mean MN frequency was calculated for each individual from 1,000 binucleated cells. Error bars represent standard deviation as calculated by the GraphPad Prism program (GraphPad Software, Inc., San Diego, CA). Values represented in the graph are the average of three experiments ± SD followed by ANOVA-Tukey's analyses to compare means.

### 2.4. Antigenotoxic Activity Using Comet Assay

Antigenotoxic activity was measured with the Comet assay [12] using human peripheral blood cells (leukocytes). Cells were preloaded with HEGM (160, 320, 640 *μ*g/mL) for 1 to 4 h, collected by centrifugation and twice washed with saline solution before being re-suspended in RPMI 1640 medium, and exposed for 5 min to H_2_O_2_ (1 mM) at 37°C. DMSO (SIGMA, 7 mM, up to 4 h) was used as negative control. Alternatively, the same procedure was done with cells preloaded with *α*-mangostin 12.5 *μ*g/mL (M3824 SIGMA) for 1 to 4 h, centrifuged, washed, and resuspended as above but exposed to H_2_O_2_ (1, 0.5 or 0.25 mM) at 37°C. Ethanol (SIGMA, 2%, up to 4 h) was used as negative control due to better solubility of the chemical. Error bars represent standard deviation as calculated by the GraphPad Prism program (GraphPad Software, Inc., San Diego, CA). Values represented in the graph are the average of three experiments ± SD followed by ANOVA-Tukey's analyses to compare means.

### 2.5. Antioxidant Activity Using* Saccharomyces cerevisiae*

Antioxidant activity of HEGM was assessed using the survival of WT* Saccharomyces cerevisiae* strain BY4742 [EUROSCARF* MATα his3Δ1 leu2Δ0 lys2Δ0 ura3Δ0*] initially exposed to HEGM (preloaded) and then plated on agar medium containing H_2_O_2_ (4 mM). Yeast media and solutions were prepared according to [15]. Complete medium (YPD) was used for routine growth of yeast cells. Stationary phase (STAT) cultures with 2 × 10^8^ cells/mL were obtained after inoculation of an isolated colony into liquid YPD and 72 h incubation at 30°C with aeration by shaking. Five mL of cell suspension was centrifuged and the collected cells were washed 3x and resuspended in 2 mL NaCl (0.9%), normalized to 10^7^ cells/mL. From this suspension, 100 *μ*L of cells was exposed to different HEGM concentrations (0 to 640 *μ*g/mL) for 0 to 4 h. Suspensions were properly diluted and plated on YPD with and without H_2_O_2_ (4 mM). Plates were incubated at 30°C for 72 h and colony numbers counted. Graphs were generated using the GraphPad Prism program (GraphPad Software Incorporation, San Diego, CA, USA); error bars represent the standard deviations of at least 3 independent experiments. The measurement of the effect of HEGM using the percentage survival versus dose with a logarithmic ordinate was performed using a typical inactivation kinetics curve [16], where the rate of decrease of the number of active single yeast cells with respect to dose is proportional to the number of active cells remaining at that dose level. When killing follows single hit kinetics, the inactivation curve is exponential, and the graph in the semilog plot will yield a linear curve with a negative slope. This presentation allows a rapid estimation of the dose reduction factor [17]. Statistical analysis between parallel experiments was performed using the standard deviation.

## 3. Results and Discussion

Many plants or their extracts used in traditional medicine have not been scientifically validated for their effectiveness and there is even less information available about the potential risks that their use might pose to public health [18]. After all, some plants used as food or for therapeutic purposes have been proven to contain substances with cytotoxic, genotoxic, or mutagenic activity [1, 3, 6, 8, 18, 19].

The pericarp of mangosteen has been used to treat constipation, diarrhea, intestinal disorders, and skin diseases [5, 17, 20]. This plant is native from Southeast Asia where the concentrate of the peel of the mangosteen is used to mainly fight infections and stomach ailments [4, 5]. Tests performed with different extractions of mangosteen pericarp (including hydroethanolic) suggested the existence of compounds with analgesic [21], antioxidant [22], and anti-inflammatory activity [23], as well as the ability to induce apoptosis in cancer cells [8], amongst other activities. However, up to date, no research has been reported investigating possible genotoxic/mutagenic activity of HEGM, the hydroethanolic extract of mangosteen pericarp.

### 3.1. Genotoxic Activity

**Figure 1:**
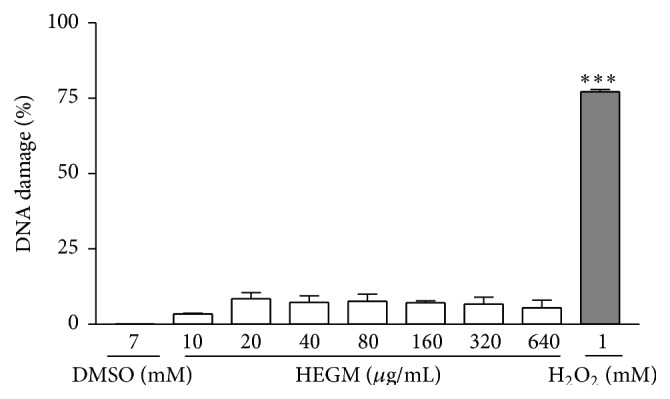
Genotoxic activity measured by Comet assay of human leukocytes: HEGM treated cells (final concentration of 10 to 640 *μ*g/mL, 1 h, 37°C), DMSO (negative control, 7 mM, 1 h, 37°C), or H_2_O_2_ (positive control, 1 mM, 1 h, 37°C). Error bars represent mean ± SD of 3 independent experiments. The analysis of variance nonparametric (ANOVA) and Tukey's test used to compare means. DMSO-induced DNA damage (basal) was subtracted from HEMG-induced DNA damage. ^*∗∗∗*^ *p* < 0.001.

**Figure 2:**
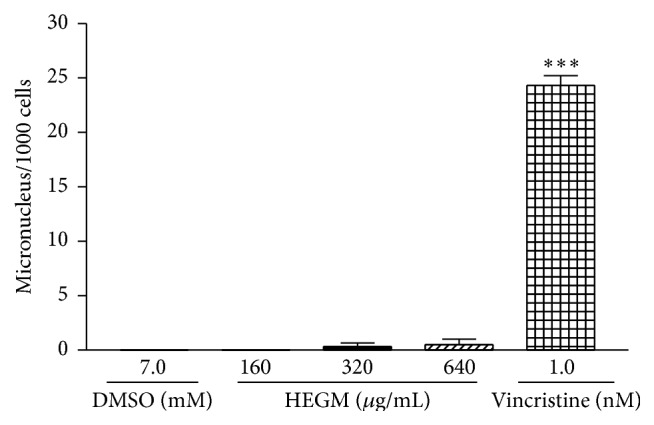
Micronuclei frequency per 1000 human lymphocytes exposed to HEGM (160 to 640 *μ*g/mL, 5% CO_2_, 37°C for 72 h), vincristine (1 nM, 5% CO_2_, 37°C for 72 h, positive control), or DMSO (7 mM, 5% CO_2_, 37°C for 72 h, negative control). The analysis of variance nonparametric (ANOVA) and Tukey test used to compare means. ^*∗∗∗*^ *p* < 0.001.

The genotoxic activity of HEGM was evaluated using the Comet assay (Figure 1), micronucleus test (Figure 2), and Salmonella/microsome assay (Table 1). HEGM was considered not mutagenic in the three test systems, as there was no statistically significant difference between untreated cells [negative control] and cells exposed to different concentrations of HEGM in the Comet assay (Figure 1); there was no statistical significant difference between the negative control and the HEGM-exposed strains in the Salmonella/microsome assay either with or without metabolization (Table 1). Finally, whereas vincristine-exposed cells exhibited a significant increase in micronucleus frequency, all other treatments, that is, negative control and exposure to HEGM, did not (Figure 2).

The Comet assay has been used to evaluate genotoxicity of extracts from* Polyalthia longifolia* [24],* Cedrela odorata* L. and* Juglans regia* L. [25], and* Acacia aroma* [26] and demonstrated their safety. On the other hand, a survey of bark extract of* Nauclea*, traditionally used in African countries for the treatment of fever [27], diarrhea, and malaria, showed a high rate of genotoxicity in the Comet assay, suggesting that ingestion of this extract is a health risk. These findings point to the importance of genotoxic risk assessment [28], that is, to determine whether plant extracts contain substances that may damage the genetic material and thus might be putative carcinogens, endangering the health of users [1, 29]. The same holds true for extracts of* Polyscias filicifolia* S. [30],* Camellia oleifera* A. [31], and* Inula viscosa* [32], which yielded positive results in the micronucleus test that is a bioindicator of mutagenicity.

The concentration range of HEGM extract was determined in experiments using* S. typhimurium* strain TA100, with and without metabolization, and cytotoxicity was observed at concentrations higher than 20 *μ*g/plate in the absence of S9 mix and 500 *μ*g/plate in the presence of S9 mix (data not shown). In the mutagenicity assay the dose range between 100 and 500 *μ*g/plate was used in the presence of S9 mix and a concentration range between 4 and 20 *μ*g/plate was applied in the absence of S9 mix (Table 1). The extract was not mutagenic in strain TA98 (detects frameshift mutation in the DNA target –C-G-C-G-C-G-C-G) regardless of absence or presence of metabolic activation. Also, no mutagenicity was detected in strain TA100 (detects base pair substitutions (leucine [GAG] by proline [GGG])) in the absence or presence of metabolic activation. This method was already used to screen the extracts of* Parthenium hysterophorus* L. [33]* Peperomia pellucida* L.,* Eichhornia crassipes* S., and* Colocasia esculenta* S. [11], where no mutagenicity at the evaluated concentrations was found. Also the Salmonella/microsome assay has been used to evaluate the biosafety of extracts of* Parthenium hysterophorus* L. [33]* Peperomia pellucida* L.,* Eichhornia crassipes* S., and* Colocasia esculenta* [11]; no mutagenicity was observed at evaluated concentrations by this effective method for the detection of genotoxicity.

### 3.2. Antigenotoxic Activity

**Figure 3:**
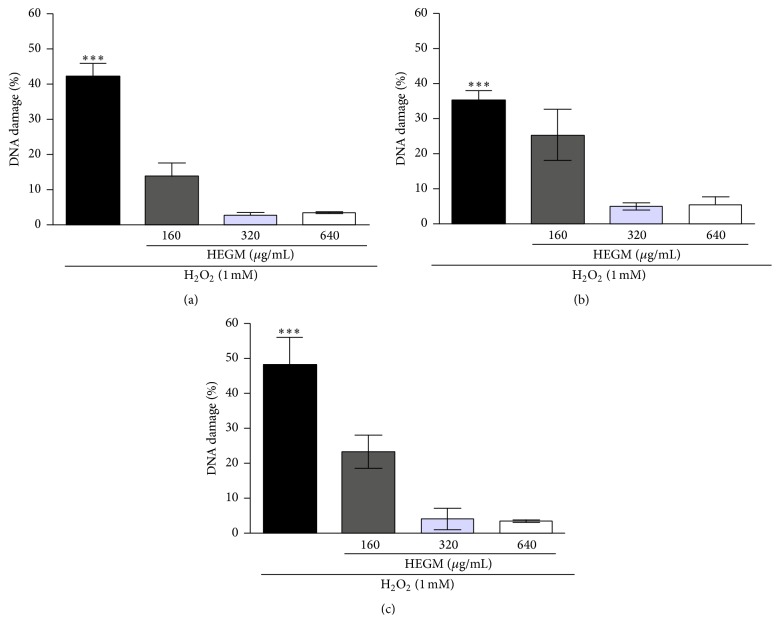
Antigenotoxic activity as measured by amount of DNA damage as detected by the Comet assay. HEGM preloaded human leukocytes (160, 320, and 640 *μ*g/mL up to 4 h); all following cells were treated with H_2_O_2_ (1 mM, 5 min, at 37°C). (a, b, and c) The cells were preloaded to HEGM concentrations for 1, 2, and 4 h, respectively, followed by exposure to H_2_O_2_ for 5 minutes. For positive control, cells were treated only with H_2_O_2_ (first column). The analysis of variance nonparametric (ANOVA) and Tukey test used to compare means, thus evaluating the reduction in DNA damage. ^*∗∗∗*^ *p* < 0.001.

We also used the Comet assay to assess the antigenotoxic activity of HEGM (Figure 3) and found it to be effective against DNA damage induced by H_2_O_2_ in doses up to 320 *μ*g/mL (1, 2, and 4 h exposure). Comparing preloading of HEGM up to 4 hours of exposure, when doubling the dose from 160 to 320 *μ*g/mL we found increased DNA-induced damage protection observable as reduced amount of detected DNA damage. However, at 640 *μ*g/mL, results were almost the same when compared to those at 320 *μ*g/mL, indicating that the lower concentration of HEGM already achieved maximum protection. Also, when comparing 2 to 4 hours of HEGM preloading, results of DNA-induced damage protection were similar. Protection of cells may be due to potential antioxidants present in HEGM [17, 18] that are sufficient to neutralize DNA damage inducing H_2_O_2_-derived free radicals [31], thus preventing DNA-strand breaks detected by Comet assay. Internal controls validated that there was no significant increase in DNA damage at any exposure time and concentration of HEGM preloading when compared to the negative control (DMSO). However, there was a significant increase when comparing positive (H_2_O_2_) to negative control (DMSO).

Indeed, it was shown that extracts of* Portulaca oleracea* L. [31],* Gymnema montanum* [32], and olive leaf [33] were effective to protect against DNA damage induced by H_2_O_2_-generated oxidative stress; similarly* Gentiana asclepiadea* extracts showed protection against oxidative damage caused by H_2_O_2_ [34] and leaf extract of* Dendrobium speciosum* contains compounds protecting against 4NQO-induced DNA damage [35]. The discovery of substances able to protect the genetic material from genotoxic agents is important as it may lead to products or procedures that may aid in the prevention of genetic damage-induced diseases such as cancer [7, 8].

**Figure 4:**
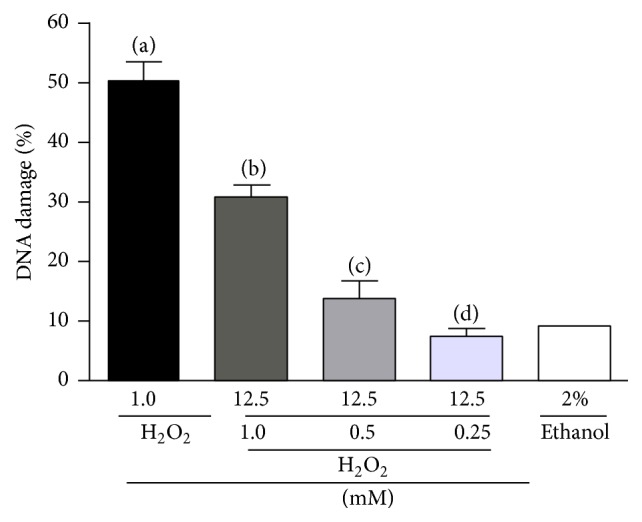
Antigenotoxicity of alpha-mangostin measured by the amount of DNA damage detected by the Comet assay. Human leukocytes were treated (preloaded) with 12.5 *μ*M alpha-mangostin for 4 h and then exposed for 5 minutes to different concentrations of H_2_O_2_ (1, 0.5 and 0.25 mM) at 37°C. (a) Positive control, 1 mM H_2_O_2_; (b, c, and d) H_2_O_2_ exposure of 1, 0.5, and 0.25 mM, respectively; ethanol as negative control. (a) versus (b, c, and d) *p* < 0.001; (c) versus (d) (*p* < 0.5).

According to HPLC measurements the HEGM extract used in this study contained 9.67%  *α*-mangostin (c.f. Supplementary Material 1 (see Supplementary Material available online at http://dx.doi.org/10.1155/2016/3430405)) which means that there is 61.44 *μ*g of *α*-mangostin (or 16.1 *μ*M) in 640 *μ*g of HEGM. In order to test whether *α*-mangostin was responsible for DNA antigenotoxicity, pure *α*-mangostin isolated from pericarp of* Garcinia mangostana* (SIGMA) was used (Figure 4). Results demonstrated that a concentration of 12.5 *μ*M *α*-mangostin protected DNA from damage caused by H_2_O_2_ in a dose dependent manner. Higher doses led to cellular cytotoxicity (data not shown). Thus the results of Figure 4 suggest that some components present in the pericarp of the* Garcinia mangostana* L., especially, *α*-mangostin, protect against H_2_O_2_-induced DNA damage.

### 3.3. Anti-Oxidant Activity

**Figure 5:**
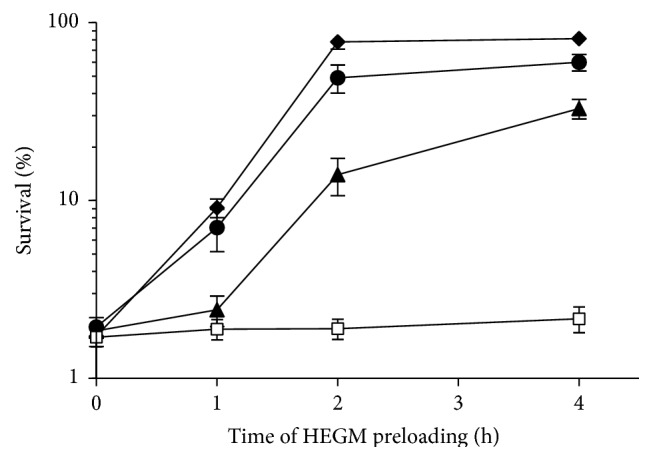
Survival of WT strain of* S. cerevisiae* to chronic H_2_O_2_ (4 mM) induced oxidative stress. Cells preloaded (up to 4 h) with either with saline (□) or with HEGM at exposure concentrations of 160 *μ*g/mL (▲); 320 *μ*g/mL (●); 640 *μ*g/mL (*◆*).

The antioxidant activity of HEGM was shown* in vitro* using the DPPH assay and results were similar to those related by Da Silva and coworkers [22] (Supplementary Material 2). We then tested HEGM for its* in vivo* antioxidant activity using* S. cerevisiae* preloaded with HEGM (from 160 to 640 *μ*g/mL) and then plating these cells on H_2_O_2_-containing agar (Figure 5). After one hour of HEGM pretreatment, there was already an increase in survival at HEGM exposure doses of 320 and 640 *μ*g/mL, and, after two hours, before loading, there was a further significant increase in survival of yeast as compared to the one-hour treatment and the negative control. Yeast cells preloaded with 320 *μ*g/mL HEGM for two hours reached about 60% survival and when again doubling the HEGM dose about 80% survival was achieved (Figure 5).

Doubling the time of exposure in preloading did not enhance protective antioxidant capacity. Taken together, these results suggest that HEGM contains at least one compound with antioxidant activity that, when taken up by the yeast cells, can neutralize the oxidant effects of H_2_O_2_, most probably that of free radicals, thus alleviating cellular oxidative stress. Preloading yeast cells with HEGM for two and four hours yielded better protection against H_2_O_2_-induced oxidative stress than shorter exposure times. Our result is in agreement with published data that suggest the presence of compounds with antioxidant activity in the pericarp of* Garcinia mangostana* L. [22].

## 4. Conclusions

In summary, the hydroethanolic extract of* G. mangostana* showed no genotoxicity/mutagenicity at exposure concentrations up to 640 *μ*g/mL but was effective as an antioxidant for yeast and effective in protecting DNA against damage from free radicals produced by H_2_O_2_.

The absence of genotoxicity together with the shown antioxidant* in vivo* properties makes application or ingestion of extracts of* G. mangostana* safe and even beneficial to humans as incidence of oxidative stress-induced genetic damage may be lowered. Contributing to prevention of DNA damage-caused disease by neutralizing free radicals derived by either cellular metabolism or external agents, mangosteen extract, or isolated active compounds thereof may thus hold promise for pharmaceutical or nutraceutical applications.

## Supplementary Material

Supplementary material contains additional data about the instrumentation and HPLC conditions; quantification of a-mangostin in G. mangostana extracts; details about measuring antioxidant activity with DPPH and cell viability test by trypan blue, as well as exemplary figures of Comet assay, micronuclei assay, antioxidant, and cell viability test.

## Figures and Tables

**Table 1:** Induction of *his+* revertants in *S. typhimurium* strains by *Garcinia mangostana* extract with and without metabolic activation (S9 mix).

*S. typhimurium* strains					
Substance	Concentration (*µ*g/plate)	TA100		TA98	
		Rev./plate^a^	MI^b^	Rev./plate^a^	MI^b^
Without metabolic activation (−S9)					
NC^c^ extract	—	95.3 ± 19.9	—	30.0 ± 5.2	—
	4	90.3 ± 10.1	0.95	28.0 ± 2.7	0.93
	8	102.0 ± 16.6	1.07	20.3 ± 4.6	0.68
	12	99.3 ± 5.03	1.04	29.0 ± 8.7	0.97
	16	95.0 ± 19.5	1.00	28.3 ± 6.8	0.94
	20	81.3 ± 57.1	0.85	19.7 ± 9.0	0.66
PC^d^	0.5 (4NQO)/1 (NaN_3_)	479.7 ± 57.1^*∗∗∗*^	**5.03**	362.7 ± 34.5^*∗∗∗*^	**12.09**

With metabolic activation (+S9)					
NC^c^ extract	—	90.3 ± 30.4	—	49.3 ± 9.2	—
	100	81.0 ± 12.3	0.90	46.3 ± 7.5	0.94
	200	82.0 ± 9.5	0.91	47.0 ± 4.6	1.14
	300	86.7 ± 15.7	0.96	51.3 ± 4.0	0.95
	400	80.3 ± 13.5	0.89	58.0 ± 13.9	0.92
	500	79.0 ± 8.7	0.88	66.0 ± 24.3	1.14
PC^d^	1 (AFB_1_)	296.3 ± 62.7^*∗∗*^	**3.28**	569.7 ± 15.7^*∗∗∗*^	**11.55**

^a^Number of revertants/plate: mean of three independent experiments ± SD; ^b^MI: mutagenic index (number of *his*+ induced in the sample/number of spontaneous *his*+ in the negative control); ^c^NC: negative control DMSO (10 *µ*L) used as solvent for the extract; ^d^PC: positive control (−S9) sodium azide to TA100; 4-NQO to TA98; (+S9) aflatoxin B_1_. ^*∗∗*^Data significantly different in relation to the negative control *p* < 0.01; ^*∗∗∗*^ *p* < 0.001.
