# Supplementary material for: DNA Protection against Oxidative Damage Using the Hydroalcoholic Extract of Garcinia mangostana and Alpha-Mangostin
Source: Evid Based Complement Alternat Med. 2016 Mar 6;2016:3430405. doi: 10.1155/2016/3430405 (PMC4799812; doi:10.1155/2016/3430405)
Supplement: Supplementary file 1 — Supplementary material contains additional data about the instrumentation and HPLC conditions; quantification of a-mangostin in G. mangostana extracts; details about measuring antioxidant activity with DPPH and cell viability test by trypan blue, as well as exemplary figures of Comet assay, micronuclei assay, antioxidant, and cell viability test. [file 3430405.f1.docx]

**Supplementary material 1**

Instrumentation and HPLC conditions (Abdalrahim et al., 2012)

The instrument consisting of Shimadzu Rapid Separation LC (RSLC) system was equipped with an auto sampler, quaternary pump, degasser, column oven, and a DAD detector. The chromatographic analysis was carried out using a reverse phase Nucleosil C18 column (5 μm, 4.6 × 250 mm). The column temperature was set at 30°C, the mobile phase was consisting of A (acetonitrile) and B (0.1% H3PO4 in water), the elution program was isocratic at 95% (A) and 5% (B) for 10 min, the flow rate was maintained at 1 mL/min, and the injection volume was 10 μL. The spectral data from the DAD-3000RS Diode Array Detector was collected at 244 nm (Suppl. Mat 1.A), and data acquisition was performed by Chromeleon software version 6.8.

Linearity

Linearity was determined by injecting 10 μL of the standard mixture in a concentration range of 1 to 64 μg/mL. The calibration curves were obtained for each individual compound by plotting the peak area versus the concentration. Regression analysis was performed in order to determine the linearity, in terms of R^2^, of the calibration graphs (Supp. Mat 1.B).

Quantification of α-mangostin in *G. mangostana* extracts

Ten microliters of *G. mangostana* extracts were injected at 100 μg/ml, and the peak area corresponding to α-mangostin was recorded. The concentration of the marker compounds in the samples was calculated by applying the linear regression equations of the standard calibration curves. The retention time was 5 minutes. Results show that the extract used in this study had 9,67% α-mangostin.

**Supp. Mat 1.A**


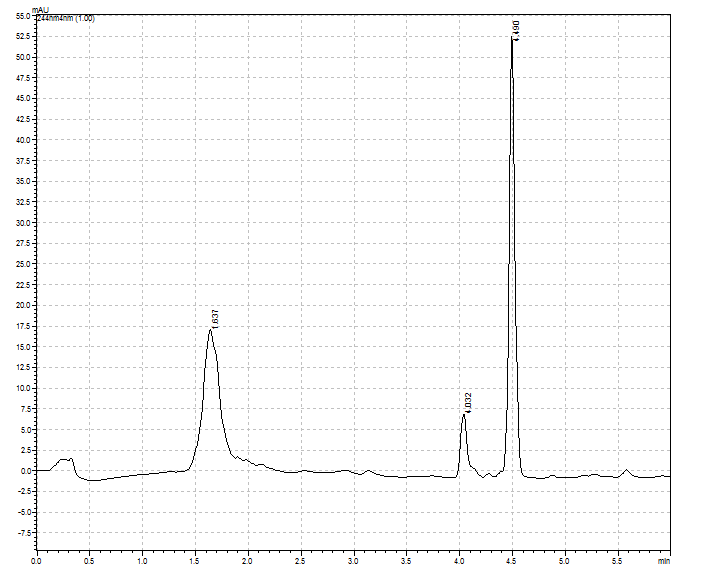


**Supp. Mat 1.B**

y = 39587x – 11144

R^2^ = 0,9994

**Supplementary material 2**

Antioxidant activity with DPPH (BRAND-WILLIAMS et al., 1995)

The antioxidant activity was determined by ability of antioxidants in HEGM to sequester stable radical DPPH•. A methanol solution of DPPH• was prepared 200 µg/ml, in order to provide absorbance at 515 nm.

The determinations were performed by adding in each well of the microplate 150 uL of the solution of DPPH• and 50 uL of methanol to the control, or the same volume for standard solutions (ascorbic acid) or concentrations of HEGM. The absorbance readings were performed after 40 min of reaction microplate spectrophotometer with incubation at 23 °C in the absence of light.

Analyses were performed in triplicate. The decrease in absorbance of the samples was correlated to control the absorbance of decay results in the percentage of kidnap free radicals.

The concentration of extract capable of capturing 50% of DPPH (IC_50_) was determined by the equation of the line. The result showed that the IC_50_ of 22.6.

**Supp. Mat 2**


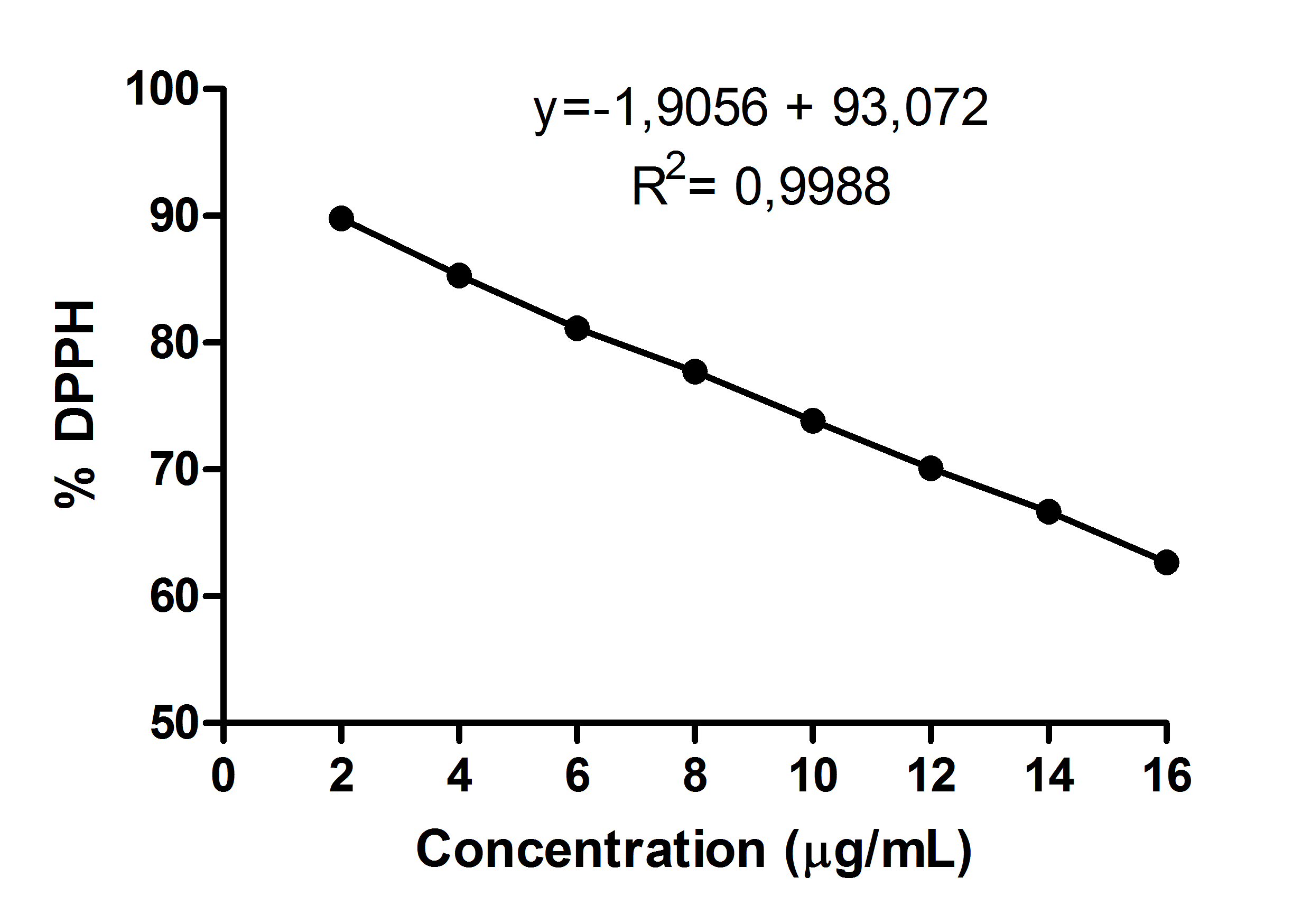


**Supplementary material 3**

Cell viability test by trypan blue CARIDDI, L et al., (2012)

Human blood cell viability test by trypan blue exclusion was used to determine adequate concentrations of HEGM. Blood was collected from a healthy individual. Samples of 20 μL of whole blood/mL of RPMI 1640 medium contained HEGM at doses ranging from 10 to 1280 μg/mL. Cell viability was measured by collecting HEGM-exposed samples after 1, 2 and 4 h of incubation at 37 °C and mixing 15 μL of each cell suspension with 15 μL of Trypan Blue (4%) in a micro-centrifuge tube. Cell suspension was analyzed by light microscopy in a Neubauer chamber. Viable cells were unstained while dead cells appeared stained blue. HEGM doses used in this study allowed cell viability >90%. Total 2,100 cells were analyzed throughout the experiment. Through the equation line IC_50_ was calculated to be 1853,42 µg/mL.


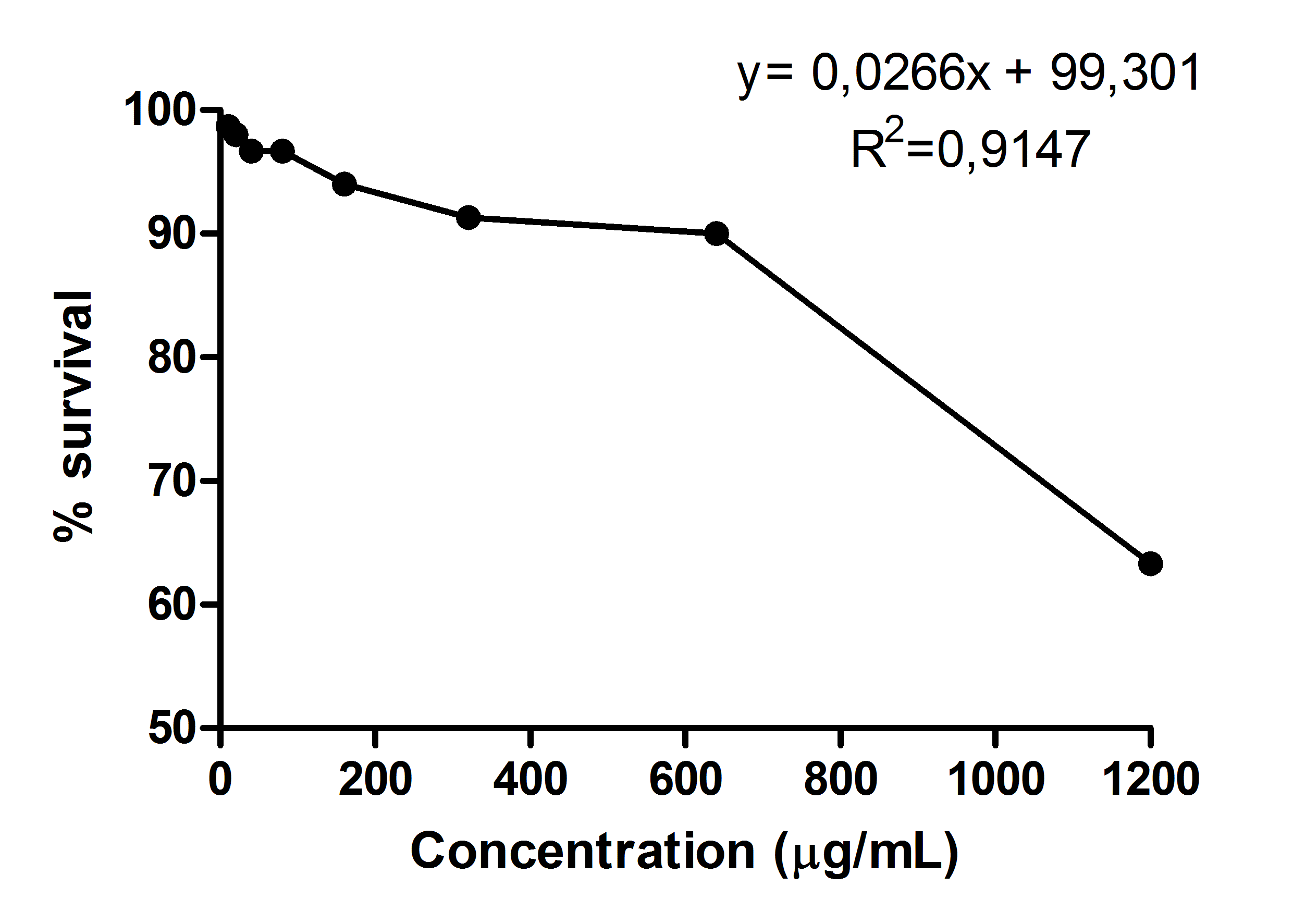


**Supplementary material 4**

Images of testing

The images shown are typical for each experiment.

**Supp. Mat 4.A**

Cell viability test by trypan blue: viable cells were unstained while dead cells appeared stained blue. This represents greater than 90% of viable cells.


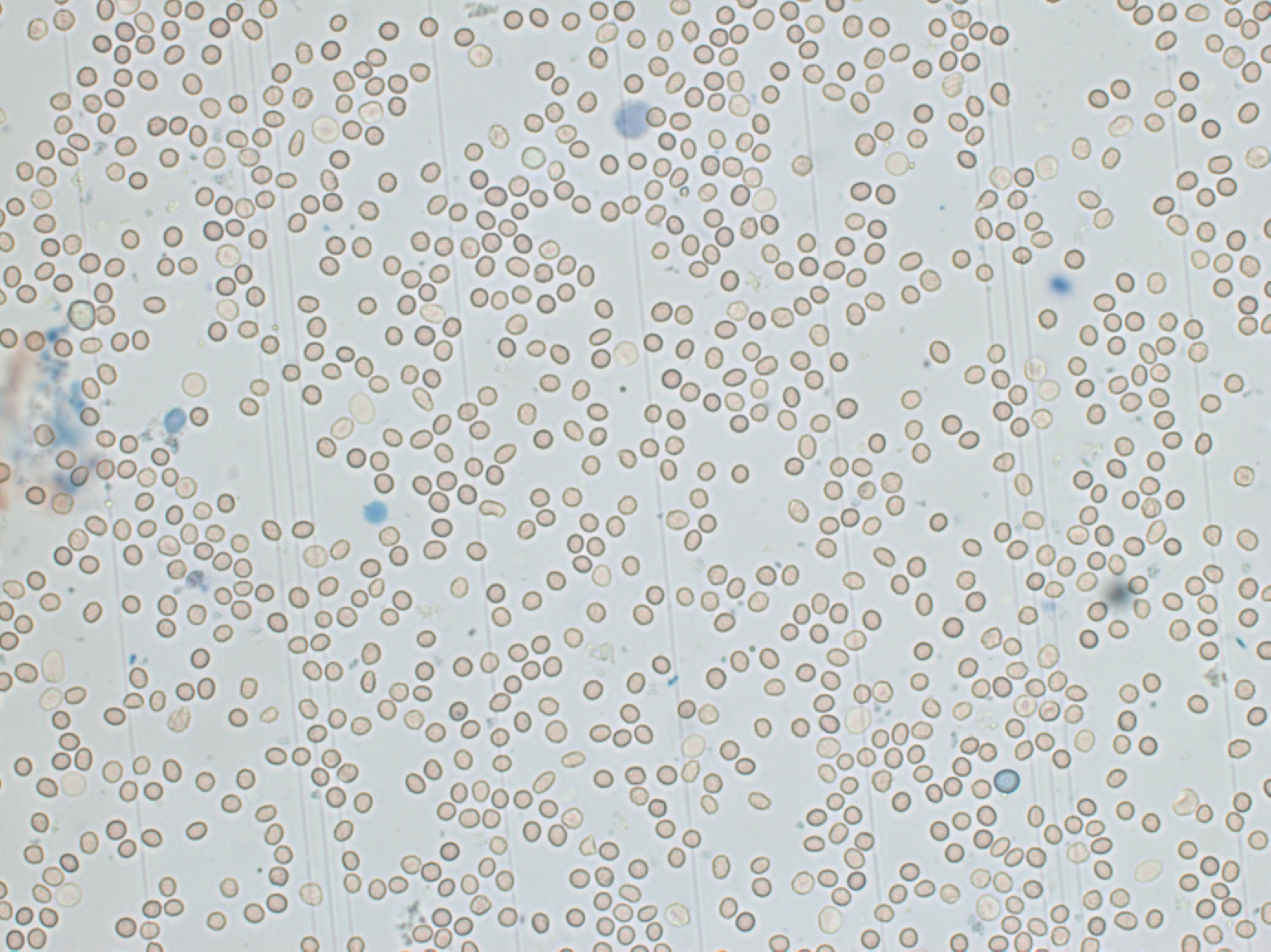


**Supp. Mat 4.B**

Comet assay

The image shown is typical for Comet assay. The figure shows different sized comets that represent different amount of chromosome breakage after treatment with hydrogen peroxide (positive control), as described in Fig. 3.


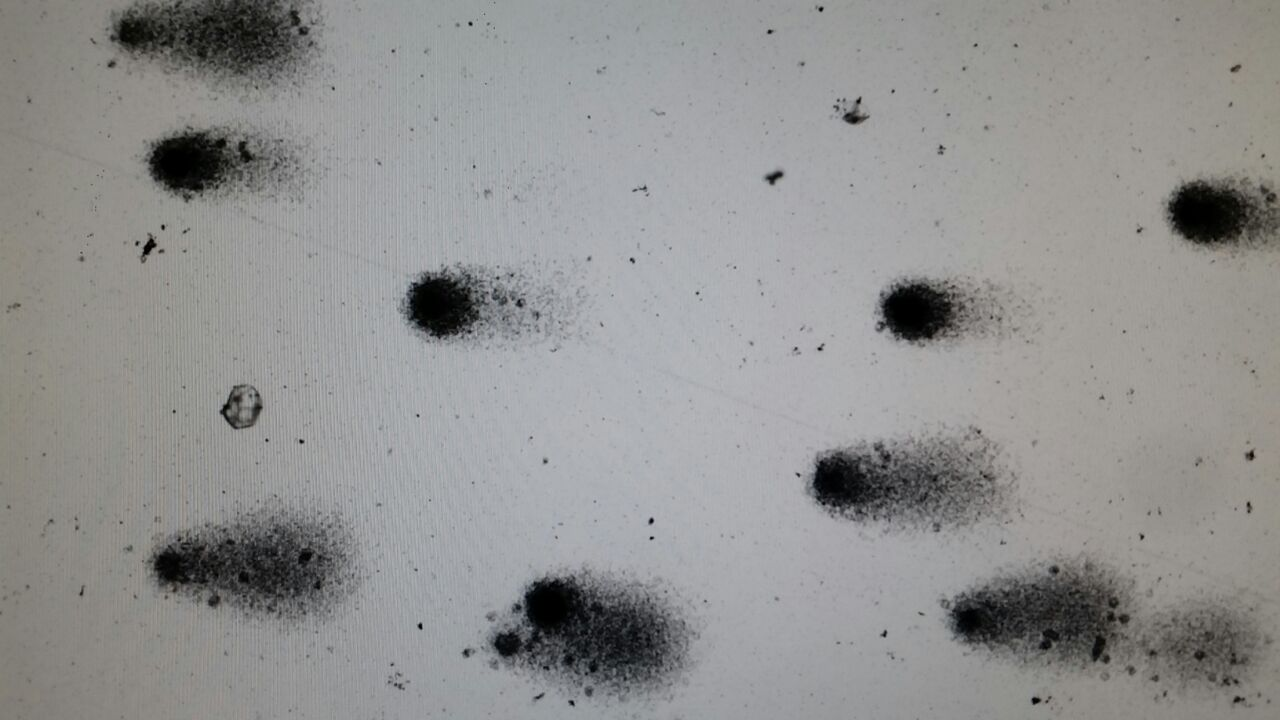


**Supp. Mat 4.C**

Micronuclei test in bi-nucleated cells

The image shown is typical for micronuclei formed after treatment with Vincristine (positive control), as described in Fig. 2.


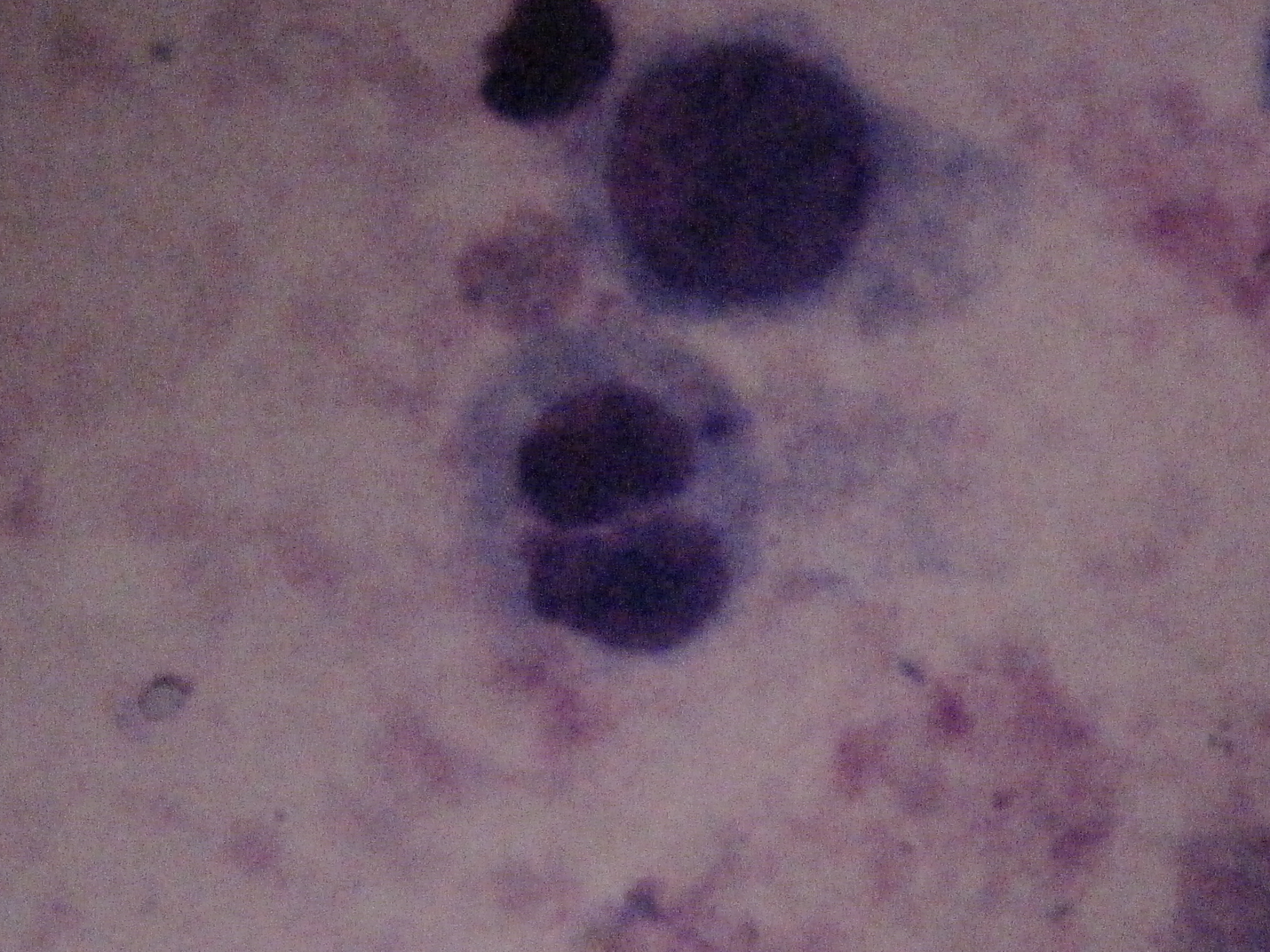


Micronuclei

**Supp. Mat 4.D**

**
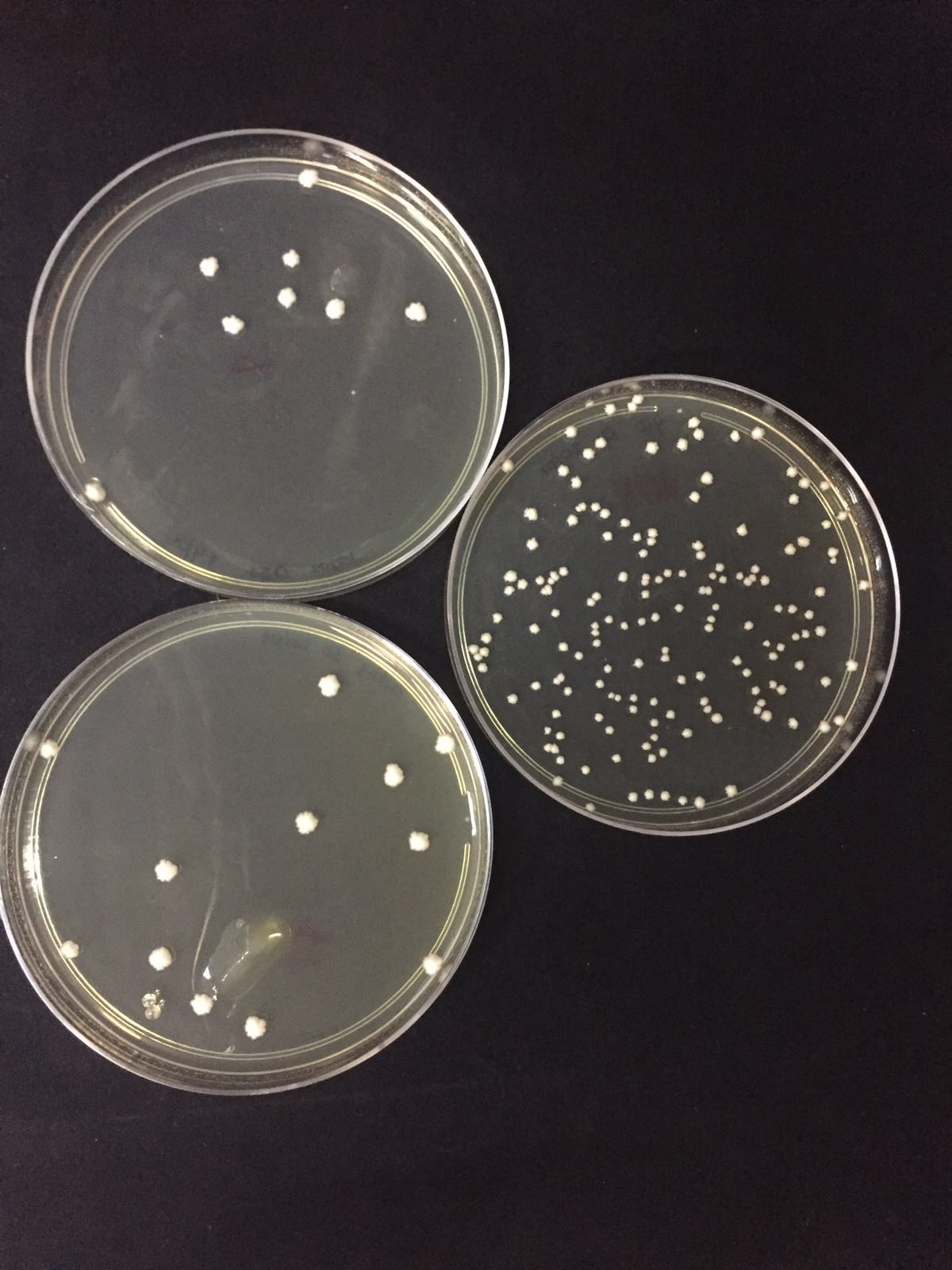

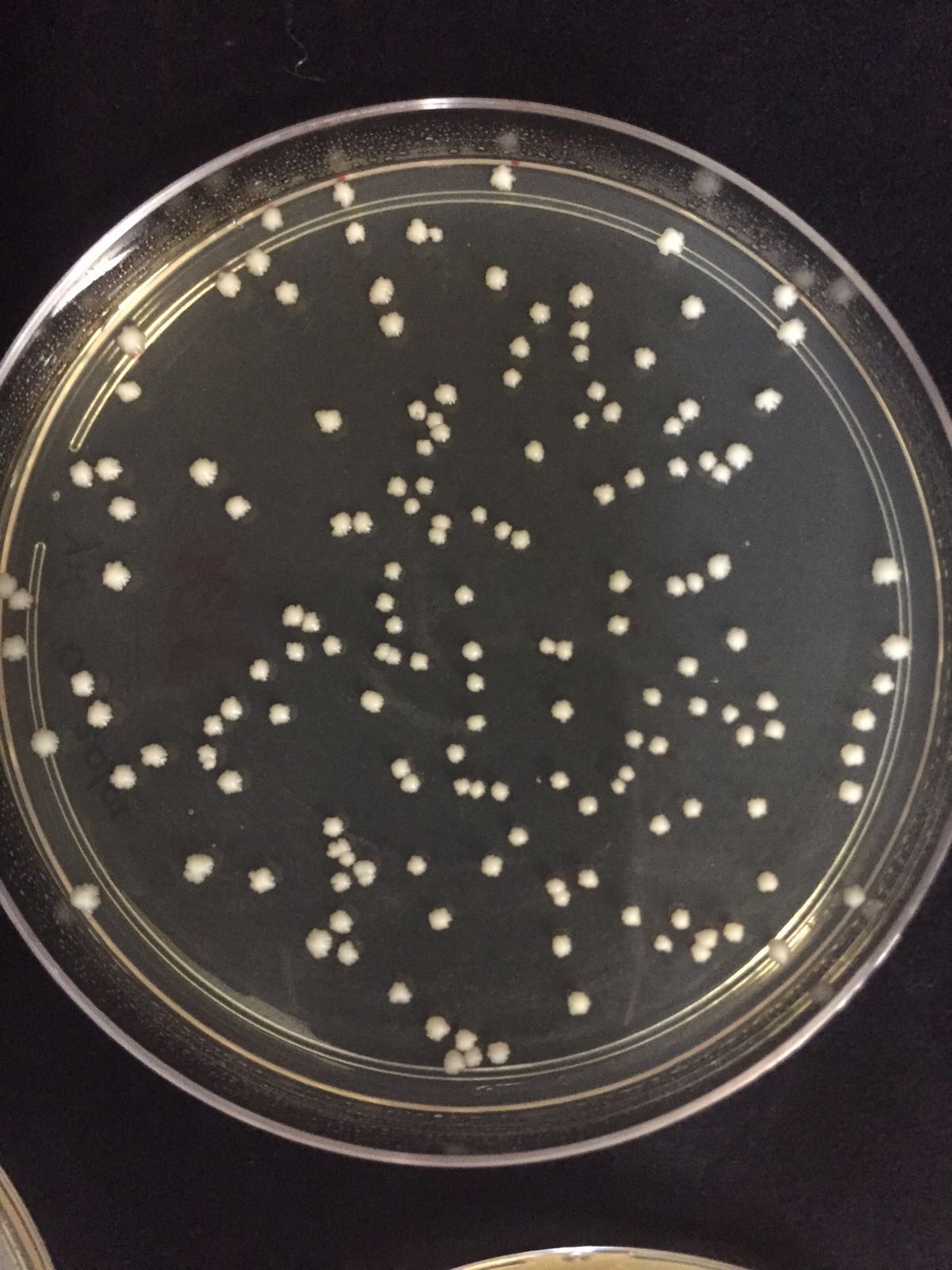
**Antioxidant assay in yeast

**Reference**

ABDALRAHIM F.A.A, KHALID M. A.S. M. J. SIDDIQUI; Z, ISMAIL, A.M. S.A. MAJID. “Quantification of α-, β- and γ-mangostin in *Garcinia mangostana* fruit rind extracts by a reverse phase high performance liquid chromatography”, *Journal of Medicinal Plants Research*, vol. 6, no 29, pp.4526-4534, 2012

BRAND-WILLIAMS, W.; CUVELIER, M.E.; BERSET, C. “Use of free radical method to evaluate antioxidant activity”. *Lebensm. Wiss - Food Science and Technology*, vol. 28, no 1, pp. 25-30, 1995.

CARIDDI, L., ESCOBAR, F., SABINI, C., TORRES, C., REINOSO, E., CRISTOFOLINI, A., SABINI, L. “Apoptosis and mutagenicity induction by a characterized aqueous extract of *Baccharis* *articulata* (Lam.) Pers (Asteraceae) on normal cells”. *Food and Chemical Toxicology*, vol. 50, no 2, pp. 155–161. (2012).
